# Outcomes of nonsuspicious contralateral nodules with active surveillance after lobectomy in patients with papillary thyroid carcinoma

**DOI:** 10.3389/fendo.2022.941080

**Published:** 2022-07-15

**Authors:** Hui Huang, Jie Liu, Xiaolei Wang, Shaoyan Liu

**Affiliations:** Department of Head and Neck Surgical Oncology, National Cancer Centre/National Clinical Research Centre for Cancer/Cancer Hospital, Chinese Academy of Medical Sciences and Peking Union Medical College, Beijing, China

**Keywords:** thyroid nodules, thyroid cancer, papillary carcinoma, thyroidectomy, active surveillance

## Abstract

**Objective:**

To observe the outcomes of nonsuspicious contralateral nodules with active surveillance in patients with papillary thyroid carcinoma (PTC).

**Methods:**

4pt?>Consecutive patients who underwent lobectomy for PTC were retrospectively reviewed. Patients with one or more nodules with nonsuspicious ultrasonography (US) features in the contralateral lobe were included.

**Results:**

Two hundred and eighty-three patients were included. All patients underwent thyroid lobectomy with ipsilateral prophylactic central neck dissection. A total of 123 patients (43.5%) were classified as ATA low-risk, and 160 patients (56.5%) were classified as intermediate-risk. The median size of the contralateral nodules was 3 mm (range, 2 to 16 mm). After a period of follow-up, the median size change of contralateral nodules was 0 mm (range, -7 to 8 mm). Eight patients (2.8%) had nodule growth >3 mm, 223 patients (78.8%) had stable or decreased nodules, and 52 patients (18.4%) had no detectable nodules. Nodules in 24 patients had suspicious US features, 16 of which were diagnosed with PTMC by either cytology after FNA (in 7 patients) or histopathology after completion thyroidectomy (in 9 patients). Another four patients received completion thyroidectomy for ipsilateral cervical lymph node metastasis. The 5-year residual lobe recurrence (RLR) rate and recurrence-free survival (RFS) rate were 7.4% and 89.8%, respectively. Multivariate analysis showed that multifocality and ATA intermediate-risk were independent predictors for RLR (HR4.083, 95%CI 1.480-11.261, *P *= 0.007; HR 6.045, 95%CI 1.370-26.662, *P *= 0.017, respectively) and RFS (HR 5.240, 95%CI 2.114-12.991, *P* < 0.001; HR 5.223, 95%CI 1.353-17.765, *P* = 0.008, respectively).

**Conclusions:**

Active surveillance for nonsuspicious contralateral nodules in patients with low-risk and selected intermediate-risk PTC is safe. Multifocality and ATA intermediate-risk are predicters for recurrence. Early detection and salvage surgery are effective.

## Introduction

A rapid rise in the incidence of thyroid cancer has been reported in recent decades (1), which is attributed mainly to the increased use of neck ultrasonography (US) (2). Thyroid cancer occurs in 5% to 15% of patients with thyroid nodules, as previously reported (3). Thyroid nodules may be detected in up to 75% of women by high-resolution US examination, and contralateral nodules are common in patients with cytologically proven thyroid cancer (4). According to the 2015 American Thyroid Association (ATA) guidelines, contralateral nodules may be a criterion for total thyroidectomy because of plans for radioiodine (RAI) therapy or to facilitate follow-up strategies or address suspicions of bilateral disease (5). The National Comprehensive Cancer Network (NCCN) guidelines also recommended total thyroidectomy for proven papillary thyroid carcinoma (PTC) with contralateral thyroid benign nodules (6).

Routine total thyroidectomy has been questioned in patients with PTC. Studies have reported that lobectomy is effective and safe. The recurrence rates for low- to intermediate-risk patients treated with lobectomy (unilateral lobectomy and/or isthmusectomy) were 4.1–5.7% in the thyroid remnant gland, 1–8.5% in regional lymph nodes, 0–3.2% in distant organs and 0–2.0% mortality (7–9). The few recurrences that develop during long-term follow-up after thyroid lobectomy can be readily detected by neck US and treated with salvage surgery without affecting survival (9, 10). Active surveillance has been suggested for papillary thyroid microcarcinoma (PTMC) due to its indolent and “subclinical” features (11, 12), leading to the ATA recommendation not to perform fine-needle aspiration (FNA) for nodules smaller than 1 cm even with suspicious US features and the option for active surveillance in patients with PTMC (5, 13).

Surgical complications, including recurrent laryngeal nerve (RLN) injury and hypoparathyroidism, are less common in patients undergoing thyroid lobectomy than in those undergoing total thyroidectomy (14–16).

Controversy exists regarding the extent of surgery for unilateral PTC with nonsuspicious contralateral nodules. Several studies have shown a high incidence of contralateral occult PTC, up to more than 40% (17, 18). However, these studies did not take into consideration the adverse clinicopathological characteristics of proven unilateral PTC, such as multifocality, large tumor size and extrathyroidal extension, and the US features of the contralateral nodules, which may guide careful detection and evaluation of occult disease. To observe the outcomes of nonsuspicious contralateral nodules with active surveillance in PTC patients treated with lobectomy (unilateral lobectomy and isthmusectomy) and guide the treatment decisions in patients with PTC and bilateral nodules, a retrospective analysis was designed and performed in a tertiary hospital.

## Materials and methods

### Study design and data collection

Eleven thousand five hundred and sixty-two consecutive patients who underwent surgery for PTC in a tertiary hospital from January 2014 to December 2017 were retrospectively reviewed, of whom, 4522 patients were operated with lobectomy and 572 patients had nonsuspicious nodule(s) in the contralateral lobe. The inclusion criteria were one or more nonsuspicious nodules in the contralateral lobe before surgery in adult patients (≥18 years) with PTC. Nonsuspicious nodules were defined as nodules with benign US features according to the thyroid imaging-reporting and data system (TI-RADS) (19). Exclusion criteria included US result unavailable, a tumor confined in the isthmus, aggressive histologic subtypes, a maximum diameter of the tumor of >4 cm, gross extrathyroidal extension (gETE), clinical cervical lymph node metastases (cN1), distant metastases (M1), prior radiation exposure of the neck or a family history of PTC. Patients with other organ malignant tumors in the same period or followed for less than 12 months were also excluded.

The extent of surgery was determined by the surgeon with consideration for patient preference. All patients underwent thyroid lobectomy (unilateral lobectomy and isthmusectomy) with ipsilateral prophylactic central neck dissection. Active surveillance was used for the contralateral nodules. All patients were treated with levothyroxine tablets and none of them were treated with iodine 131. The data, including demographics, US features of nodules, surgical reports, histopathological characteristics and outcomes, were obtained from the medical records. Hashimoto’s thyroiditis was determined on the basis of the histopathological examination. Staging was performed according to the American Joint Committee on Cancer TNM Stage for Thyroid Cancer (8th Edition, 2017) (20). The initial risk stratification was performed according to the 2015 American Thyroid Association (ATA) guidelines (5).

### Follow-up and statistical analysis

The follow-up period was defined as the time between the initial surgery and the last visit. Physical examination and neck US were performed biannually. Chest CT scans or X-radiography were performed every year. Thyroglobulin was not routinely measured. The main endpoints of this study were residual lobe recurrence (RLR) and recurrence-free survival (RFS). Recurrence in the contralateral lobe or cervical lymph node was diagnosed by FNA cytology or histopathology. Nodule growth was defined as a change of >3 mm in size. For multiple nodules, the change in size was documented for the dominant nodule. The median follow-up time was 51 months (range, 12 to 99 months).

SPSS v20.0 (SPSS Inc., Chicago, IL, USA) software was used for statistical analysis. Associations between two categorical variables were examined using the x2 test and Fisher’s exact test. Survival data were analyzed by means of the Kaplan–Meier method, and survival curves were compared using the log-rank test on univariate analysis. Cox regression analysis was used for multivariate analysis. All statistical tests were two-sided, and *P <*0.05 was considered statistically significant. This was a retrospective analysis and would not cause any harm to the patient, informed consent was not obtained from the patients, and the study was approved by the institutional ethics committee.

## Results

### Characteristics of the patients and PTC

Two hundred and eighty-three patients were included in this study. The median age was 44 years (range, 18 to 75 years), and 42 (14.8%) patients were ≥ 55 years old. A total of 216 (63.7%) patients were female. The median tumor size was 0.7 cm (range, 0.2 to 2.9 cm), and 21.2% of patients had a tumor larger than 1 cm. Most patients had classic (67.1%) and follicular variant (31.1%) PTC. Seventy-eight patients (27.6%) had multifocal disease, and 141 patients (49.8%) had microscopic ETE (mETE). Fifty-five (19.4%) patients had a background of Hashimoto’s thyroiditis. A total of 121 (42.8%) patients had pathological lymph node metastasis in ipsilateral central neck (pN1a). The median number of positive lymph nodes was 2 (range, 1-11). Two hundred and seventy-seven patients (97.9%) were classified as stage I, and six patients (2.1%) were classified as stage II. According to the 2015 ATA risk stratification system, 123 patients (43.5%) were classified as low-risk, and 160 patients (56.5%) were classified as intermediate-risk (**Table 1**).

**Table 1:** Baseline characteristics.

Characteristic		N
Total number of patients		283
Median age, y (range)		44 (18–75)
Sex, n (%)	Female	216 (67.3)
	Male	67 (23.7)
Age group, n (%)	< 55 y	241 (85.2)
	≥ 55 y	42 (14.8)
**PTC in Lobectomy**		
Median PTC size, cm (range)		0.7 (0.2-2.9)
PTMC	No	60 (21.2)
	Yes	223 (78.8)
PTC variant, n (%)	Classic	190 (67.1)
	Follicular variant	88 (31.1)
	Oncocytic	4 (1.4)
	Encapsulated	1 (0.4)
Multifocality, n (%)		78 (27.6)
mETE, n (%)		141 (49.8)
Hashimoto’s thyroiditis, n (%)		55 (19.4)
pT stage, n (%)	1a	223 (78.8)
	1b	51 (18.0)
	2	9 (3.2)
pN stage, n (%)	0	162 (57.2)
	1a	121 (42.8)
AJCC 8 stage, n (%)	I	277 (97.9)
	II	6 (2.1)
ATA risk group	low	123 (43.5%)
	intermediate	160 (56.5%)
**Nodules in contralateral Lobe**		
Median nodule size, mm (range)		3 (2–16)
Multiple nodules, n (%)		165 (58.3)
US echo patterns, n (%)	anechoic	53 (18.7)
	hypoechoic	133 (47.0)
	isoechoic	4 (1.4)
	mix-echoic	92 (32.5)
	hyperechoic	1 (0.4)
TI-RADS category	2	273(96.5)
	3	10(3.5)

### Characteristics of the contralateral nodules

The median size of the contralateral nodules was 3 mm (range, 2 to 16 mm). More than one nodule was seen on US in 165 (58.3%) patients. Sonographic patterns included anechoic (18.7%), hypoechoic (47.0%), isoechoic (4.0%), mix-echoic (32.5%) and hyperechoic (0.4%). According to the TI-RADS system, contralateral nodules in 273 patients were classified as TI-RADS 2, and 10 patients were classified as TI-RADS 3, all of which were defined as nonsuspicious nodules, and none were assessed by FNA before initial surgery.

### Outcomes of the contralateral nonsuspicious nodules

After a period of follow-up, the median size change of contralateral nodules was 0 mm (range, -7 to 8 mm). Eight patients (2.8%) had nodule growth > 3 mm in size, 223 patients (78.8%) had stable or decreased nodules, and 52 patients (18.4%) had no detectable nodules (**Table 2**). The nodule growth ratio was only 2.8%. Twenty-four patients had suspicious US features according to the TI-RADS system (2 had a growth >3 mm), seven of whom received FNA and were cytologically diagnosed with PTC. The median size of the cytologically diagnosed tumor was 4 mm (range, 3 to 6 mm). Nine of the patients with suspicious US features received completion thyroidectomy and were pathologically diagnosed with PTC, all of whom had PTMC confined to the thyroid gland with a median size of 5 mm (range, 4 to 9 mm), and four had multifocal PTC. Three patients had contralateral central lymph node metastasis, and one had ipsilateral cervical lymph node metastasis. Overall, 16 patients were diagnosed with RLR. The median time to recurrence was 28 months (8–74). The patient with ipsilateral cervical lymph node metastasis received RAI therapy (100 mCi) after completion surgery.

**Table 2:** Characteristics of nodules in contralateral lobe after a period of follow-up.

Characteristic		N
**TI-RADS category**, **n (%)**	1	52 (18.4)
	2	201 (71.0)
	3	6 (2.1)
	4a	2 (0.7)
	4b	6 (2.1)
	4c	4 (1.4)
	5	3 (1.1)
	6	9 (3.2)
**Median nodule size, mm (range)**		3 (0 – 18)
**Median growth size, mm (range)**		0 (–7 – 8)
**Nodules Growth ratio**		8/283 (2.8%)
**Nodule status in contralateral lobe**		
**Size change, n (%)**	No nodule	52 (18.4)
	Unchanged	223 (78.8)
	Growth	8 (2.8)
**Suspicious malignancy, n (%)**		8 (2.8)
**Diagnosed malignancy, n (%)**		16 (5.6)
**Completion thyroidectomy for ipsilateral LNM**		4 (1.4)

Another four patients received completion thyroidectomy for ipsilateral cervical lymph node metastasis, and no disease was found in the residual thyroid lobe by histopathological examination. The median time to ipsilateral cervical lymph node metastasis was 31.5 months (12–44). All patients received RAI therapy (range, 30-100 mCi) after salvage surgery.

No patient who received completion thyroidectomy had permanent RLN injury or permanent hypoparathyroidism.

### Predicters for residual lobe recurrence

The 5-year RLR rate was 7.4%. Univariate analysis showed that sex (*P* = 0.653), age group (*P* = 0.860), PTMC (*P* = 0.392), mETE (*P *= 0.292), Hashimoto’s thyroiditis (*P *= 0.738) and pN stage (*P* = 0.135) were not associated with RLR. Hypoechoic nodules had a higher RLR rate than nodules with other echo patterns, but the difference was not significant (*P* =0.061). Multifocality and ATA intermediate-risk were significantly associated with a higher RLR rate (*P* = 0.003 and 0.009). Multivariate Cox regression analysis showed that multifocality [hazard ratio (HR) 4.083; 95% confidence interval (CI) 1.480-11.261; *P *= 0.007] and ATA intermediate-risk [hazard ratio (HR) 6.045; 95% confidence interval (CI) 1.370-26.662; *P *= 0.017] were both independently associated with increased RLR (**Table 4**).

### Predicters for recurrence-free survival

No distant metastasis occurred. All patients survived free of disease. The 5-year RFS rate was 89.8%. Univariate analysis showed that sex (*P* = 0.824), age group (*P* = 0.990), PTMC (*P* = 0.828), ultrasonic echo (*P* = 0.362), mETE (*P* = 0.241) and Hashimoto’s thyroiditis (*P* = 0.736) were not associated with RFS. Multifocality (*P* = 0.000), ATA intermediate-risk (*P* = 0.004) and pN stage (*P* = 0.035) were significantly associated with RFS (**Table 3**). Multivariate Cox regression analysis showed that multifocality (HR 5.240; 95% CI 2.114-12.991; *P* < 0.001) and ATA intermediate-risk (HR 5.223; 95% CI 1.353-17.765; *P* = 0.008) were both independently associated with decrease RFS (**Table 4**).

**Table 3:** Univariate analysis of predicters for RLR and RFS.

Characteristics		RLR rate (%,5-ys)	*P* value	RFS rate (%,5-ys)	*P* value
**Sex**	Female	7.6	0.653	90.5	0.824
	Male	8.2		85.5	
**Age group**	< 55 y	7.1	0.860	90.5	0.990
	≥ 55 y	9.7		85.5	
**PTMC**	Yes	5.4	0.392	91.0	0.828
	No	15.3		84.6	
**Ultrasonic echo**	anechoic	6.0	0.061	88.5	0.362
	hypoechoic	10.9		88.1	
	isoechoic, mix-echoic, hyperechoic	3.4		92.2	
**Multifocality**	Yes	16.8	0.003	75.1	0.000
	No	3.8		95.7	
**mETE**	Yes	11.5	0.292	85.1	0.241
	No	2.5		94.3	
**Hashimoto’s thyroiditis**	Yes	7.5	0.738	90.8	0.763
	No	7.5		89.4	
**pN stage**	0	6.7	0.135	92.0	0.035
	1a	8.5		86.7	
**ATA risk group**	low	1.3	0.009	97.1	0.004
	intermediate	12.1		84.1	

**Table 4:** Multivariate analysis of the predictive factors for RLR and RFS.

Predictors	RLR			RFS		
	P value	HR	95%CI	P value	HR	95%CI
**Multifocality**	0.005	4.338	1.575-11.946	<0.001	5.240	2.114-12.991
**ATA intermediate-risk**	0.017	6.045	1.370-26.662	0.008	5.223	1.535-17.765

## Discussion

Indications for bilateral total thyroidectomy according to 2015 ATA guidelines (5) and NCCN guidelines (6) include tumor size larger than 4 cm, ETE, cN1, distant metastases, and prior radiation exposure of the neck. Total thyroidectomy is also considered for bilateral nodularity. However, total thyroidectomy for unilateral PTC with nonsuspicious contralateral nodules is controversial.

Our study describes the outcomes of nonsuspicious contralateral nodules with active surveillance after lobectomy. After a period of follow-up, only 2.8% of nodules grew more than 3 mm in size, and most patients had stable or decreased nodules. A study reviewed the natural history of nonsuspicious contralateral nodules in one hundred and twelve patients with PTC after lobectomy (21). After a median follow-up of 6 years, twenty-six nodules (23%) increased by more than 3 mm in size. Durante et al. (22) reported a growth rate of 15.4% in patients with benign nodules followed for more than 5 years. The low growth ratio in our study may be due to the short follow-up time (median 51 months). On the other hand, it may be related to the US features of the nodules in our study. Most of the nodules were small (half of the nodules were no more than 3 mm in size), and more than 51% of the nodules were anechoic (cystic) or mixed-echoic (cystic solid) nodules. Over time, cystic fluid absorption and nodule fibrosis may occur, and these nodules may shrink or even disappear under US examination. Ritter A et al. (21) reported that 41% of nodules had a stable size, 26% of nodules decreased in size, and the nodule was not present in 16 patients (14%) at the last US examination. In our study, nodules were not detected in 52 (18.4%) patients. Most nodules would not grow in size with active surveillance.

Most nodules do not cause concern as long as there are no suspicious US features, even for those with an increase in size. Growth in size is not a major predictor of malignancy (23). Further evaluation is needed, such as FNA and cytology, for nodules with suspicious US features. In our study, 24 patients (8.4%) had suspicious features according to the TI-RADS system after a period of surveillance, and 16 patients were diagnosed with PTMC by cytology or histopathology. The 5-year RLR rate was 7.4%. Studies have reported that recurrence rates after thyroid lobectomy in properly selected patients range from 1% to 4.1% (10, 14, 24). Ritter A et al. reported that the natural history of contralateral nodules after lobectomy and showed a contralateral PTC rate of 5% (21). In the literature, most patients treated with lobectomy had normal glands in the contralateral lobe. Another reason for the relatively high RLR rate in our study may be that we did not perform FNA for contralateral nodules before surgery, and some “subclinical” or “small” cancers may have been omitted. Four patients with ipsilateral cervical lymph node metastasis received salvage surgery and RAI therapy and survived free of disease. No distant metastasis or death occurred.

According to the ATA guidelines and NCCN guidelines, contralateral nodules may be an indication for a bilateral procedure (5, 6). Several recent studies demonstrated that follow-up after lobectomy is safe and effective in detecting clinically significant recurrences (9, 24) even in patients with contralateral nodules (21). The RFS for selected patients (low-intermediate risk) treated with lobectomy was comparable to those treated with total thyroidectomy (9, 10, 14). Ma T et al. compared outcomes after lobectomy in patients with benign or nonsuspicious contralateral nodules to those without contralateral nodules. The 5-year contralateral lobe recurrence-free survival rates and locoregional RFS rates were comparable (97.4% and 96.8%, 97.8% and 98.4%, respectively). Contralateral nodules alone should not be an indication for total thyroidectomy (25).

Multifocal disease is accompanied by more ETE and regional and distant metastasis than unifocal disease in PTC (26, 27). Some studies have reported that multifocal disease is a risk factor for recurrence and requires more aggressive management (28, 29). For patients with ATA low-risk PTC, the ATA guidelines provide an estimated risk of structural recurrence: unifocal PTC ≤ 1 cm (1%–2%), multifocal PTC ≤ 1 cm (4%–6%), and intrathyroidal PTC 2 cm-4 cm (~5%) (5). Size and macroscopic multifocality may play a more important role in recurrence. In current study, multifocality was an independent predictor for both RLR and RFS by univariate and multivariate analyses.

Multifocality in our study was diagnosed by histopathological examination after initial surgery, and details on the size and “macro” or “micro” could not be obtained. Nevertheless, we suggest stricter monitoring after lobectomy for unilateral multifocal PTC. A recent study by Harries V et al. showed that thyroid lobectomy alone is safe with rates of contralateral lobe PTC, regional recurrence, and overall survival comparable to those of total thyroidectomy in T1-T2 N0M0 unilateral multifocal PTC patients (30). ATA intermediate-risk group was also an independent predictor for RLR and RFS in this study. Patients stratified as ATA intermediate-risk had high risk factors such as microscopic ETE, vascular invasion or high-volume lymph node metastasis, all of which are risk factors for high rate of lymph node metastasis and tumor recurrence as demonstrated in the literatures. In current study, though with lack of data regarding the vascular invasion and aggressive histologic subtypes, all patients survived free of tumor after a re-operation for the recurrence in the residual thyroid lobe or regional lymph node. Nevertheless, we suggest a strict surveillance strategy for patients with multifocal or ATA intermediate-risk PTC, to facilitate early detection of recurrence and salvage operation in time.

The risk of complications after total thyroidectomy is greater than after lobectomy, including permanent RLN injury and permanent hypoparathyroidism, even when performed by high-volume thyroid surgeons (15, 16). Given that active surveillance of contralateral nodules by high-resolution ultrasound, early detection of recurrence and salvage surgery are effective, lobectomy may be an appropriate strategy for unilateral PTC with nonsuspicious contralateral nodules. In our study, recurrence in the contralateral residual gland and local regional lymph nodes were all detected in time and successfully dissected by salvage surgery. The extent of the initial surgery should be decided by the surgeon and each patient, considering complications of surgery, risks of recurrence and the monitoring strategy after surgery.

The relatively short follow-up time may result in an underestimation recurrence, and together with the small volume of included patients, the conclusions may be limited. As a retrospective study, given the different ideas of individual surgeons and patients’ preferences, selection bias cannot be avoided. Data regarding the total number and size of each contralateral nodule were not available, and details on the size and “macro” or “micro” of multifocal PTC could not be obtained. We were not able to analyze whether these certain factors may affect recurrence. Despite these limitations, we conclude that active surveillance for nonsuspicious contralateral nodules in patients with low-risk and selected intermediate-risk unilateral PTC is safe. Multifocality and ATA intermediate-risk are predicters for recurrence in the remnant thyroid gland and locoregional lymph node. Early detection by US and salvage surgery is effective. To obtain a more reliable conclusion, rigorous clinical trials with large volume cases and long-term follow-up are needed.

## Data availability statement

The raw data supporting the conclusions of this article will be made available by the authors, without undue reservation.

## Ethics statement

The studies involving human participants were reviewed and approved by the institutional ethics committee of National Cancer Centre/National Clinical Research Centre for Cancer/Cancer Hospital, Chinese Academy of Medical Sciences and Peking Union Medical College. Written informed consent for participation was not required for this study in accordance with the national legislation and the institutional requirements.

## Author contributions

HH and SL contributed to conception and design of the study. HH organized the database and performed the statistical analysis. HH wrote the first draft of the manuscript. HH and JL wrote sections of the manuscript. XW, SL contributed to manuscript revision. All authors read and approved the submitted version.

## Conflict of interest

The authors declare that the research was conducted in the absence of any commercial or financial relationships that could be construed as a potential conflict of interest.

## Publisher’s note

All claims expressed in this article are solely those of the authors and do not necessarily represent those of their affiliated organizations, or those of the publisher, the editors and the reviewers. Any product that may be evaluated in this article, or claim that may be made by its manufacturer, is not guaranteed or endorsed by the publisher.
